# Artificial Intelligence Use in Medical Writing: On the Meaning and Limits of Disclosure

**DOI:** 10.31662/jmaj.2026-0121

**Published:** 2026-06-12

**Authors:** Shigeki Matsubara

**Affiliations:** 1 Department of Obstetrics and Gynecology, Jichi Medical University, Shimotsuke, Tochigi, Japan; 2 Department of Obstetrics and Gynecology, Koga Red Cross Hospital, Koga, Ibaraki, Japan; 3 Medical Examination Center, Ibaraki Western Medical Center, Chikusei, Ibaraki, Japan

**Keywords:** artificial intelligence, disclosure, guideline, journal, regulation

## Abstract

This opinion paper focuses on disclosure of artificial intelligence (AI) use in medical writing; AI use beyond manuscript writing is outside its scope. Almost all journals require full disclosure of AI use. However, several barriers may hinder such disclosure. First, there is a lack of clear statements by journals on how disclosure is evaluated. Authors may be concerned that a linguistically polished manuscript accompanied by AI disclosure might be scored lower. Conversely, a journal might question the nonuse of AI assistance when a manuscript presents highly significant data written in uncomfortable English. Authors may fear that either AI “use” or “nonuse” could result in disadvantage. Second, criteria for disclosure remain unclear. Many journals require disclosure when AI is used to “create, review, revise, or edit” but not when it is used for “checking grammar, spelling, and similar tasks.” However, the distinction between “edit” and “grammar check” is a delicate one; prompts such as “edit” and “check grammar” may produce almost identical revisions. Third, it may become difficult to distinguish between one’s original expressions and AI-aided revisions after several rounds of linguistic consultation. Authors may regard the final text as their own authorship rather than AI intervention. These three factors, intentionally or unintentionally, may prevent honest and full disclosure. Journals may consider stating two points. First, that the use or nonuse of AI tools does not affect acceptance or rejection, provided that such use adheres to journal guidelines and is accurately disclosed. Second, that disclosure information is accumulated as a resource for future analysis, contributing to a more transparent publication environment. Such clarification could reduce hesitation surrounding disclosure. Transparency, from both authors and journals, would become an asset to the medical community.

## Artificial Intelligence (AI) Has Already Entered Medical Writing

I focus here on the use of AI in medical writing. AI use in medical publication beyond writing, such as concept development, interpretation, and image creation, is outside the primary scope. Recent large-scale vocabulary analysis showed that at least 13.5% of PubMed-indexed abstracts in 2024 have traits consistent with the use of large language models (LLMs; e.g., ChatGPT) ^(1)^. Given the rapid integration of AI into medical writing, this percentage will likely increase.

Journals (including *JMA Journal* [*JMAJ*]), publishers, and international organizations provide guidelines on AI use in medical writing. Although their contents vary, all consistently request “disclosure of AI use” ^(2)^. It is the authors’ responsibility to adhere to these guidelines and to declare AI use honestly. However, several barriers may hinder such disclosure. Here, I briefly summarize these barriers from an author’s perspective and ask stakeholders to consider them. The current disclosure framework assumes that authors can recognize and report AI contributions; however, this may not always hold in practice.

## Authors Are Not Notified How AI Use Affects Evaluation

How journals evaluate AI use remains unclear. Guidelines from international organizations—the Committee on Publication Ethics, the International Committee of Medical Journal Editors, and the World Association of Medical Editors—which the *JMAJ* guideline hyperlinks ^(2)^, do not address this issue. A guideline from an influential publisher ^(3)^ states, “The final decision about whether use of a generative AI tool is appropriate or permissible in the circumstances of a submitted manuscript or a published article lies with the journal’s editor.” In its introductory section, however, it also states, “We welcome the thoughtful use of AI tools.”

Let us assume two scenarios: (1) a linguistically polished manuscript, with or without a disclosure of AI use for “language editing”; and (2) a well-structured paper, with or without AI assistance for “text restructuring.” Reviewers prioritize scientific significance; however, the manner of expression inevitably influences judgment. Whether AI use might incur implicit negative bias remains unclear. Conversely, if a manuscript presenting highly significant data is written in uncomfortable English, reviewers and editors might question the nonuse of LLMs, much as they might question the nonuse of a spellchecker. Thus, although empirical evidence, to my knowledge, is lacking, it is reasonable to assume that authors may be concerned that either AI “use” or “nonuse” may result in disadvantage. Such uncertainty may discourage transparent disclosure. Because authors differ in the weight they assign to writing quality ^(4)^, and because reviewers and editors likewise vary in this regard, the evaluative implications of AI use become even more complex. Making matters more complicated, there is no “100% correct” English: correct grammar may be present, but natural English may not be. Evaluation of linguistic issues cannot be free from subjectivity.

In this opinion paper, various terminologies including “language editing,” “linguistic refinement,” and “grammar checking” are used; this follows the wording of individual journal guidelines and cited sources under discussion. The variation itself reflects the ambiguity addressed here. Thus, I intentionally retain this heterogeneity of terms.

## Criteria for Declaration or Nondeclaration Are Unclear

Of articles across 13 JAMA Network journals during the most recent examination period (August–October 2025), author-declared AI use was observed in approximately 6%; it was most commonly reported for “refinement of language” ^(5)^.

The term “refinement of language” remains ambiguous. The JAMA author guideline requests disclosure when AI is used to “create, review, revise, or edit” manuscript content, while exempting its use for “checking grammar, spelling, and similar.” This distinction aligns with policies adopted by many journals. In practice, however, the boundary is not clear.

When prompted simply to “check grammar,” contemporary AI tools often introduce native-preferred paraphrasing rather than limiting themselves to mechanical correction. In an informal comparison, I found that outputs generated by the prompts “check grammar, spelling, and similar” and “edit manuscript” were nearly identical. The practical boundary between AI use that should be disclosed and that which need not be declared therefore appears blurred.

Furthermore, even when one instructs LLMs to limit their role to “checking grammar,” they often suggest broader improvements, such as stating, “I can help improve clarity and flow.” Few authors may be able to resist this broader assistance particularly less experienced writers or nonnative English speakers, who may also be those most likely to rely on AI for manuscript preparation. An initial prompt to “check grammar” may thus evolve into substantial textual revision; yet, the author may still perceive the process as mere proofreading and choose not to disclose AI use.

## One May Not Be Able to Discern between One’s Original and AI-Aided Expressions

Authors themselves may sometimes encounter the following situation: after repeated rounds of revision and discussion with AI, they can no longer clearly distinguish between their original wording and AI-generated or AI-modified expressions. For example, I typically revise my manuscripts approximately 20 times and recommend that my staff do the same. Before the AI era, after numerous revisions without external textual intervention, one naturally recognized the final version as one’s own. When AI becomes involved in the process—one writes, AI edits, one revises, and AI modifies—and this cycle is repeated many times, it may become difficult to determine what proportion of the final text is genuinely one’s own. Although no empirical evidence currently addresses this phenomenon, one may gradually develop the subjective sense that “I wrote this.”

This situation is not entirely new. When I studied cell biology, I drafted sentences daily and discussed them with a senior researcher who carefully edited my work. When the manuscript was completed, although I remained fully aware of his assistance, I nonetheless felt that my “own” effort had taken shape as a paper. A similar experience occurs in childhood memory: after hearing a story repeatedly, one may come to feel as though one personally remembers the event. Distinguishing authentic memory from constructed memory is not always straightforward. I here refer to the latter as “pseudo-memory,” used metaphorically (not a formal term).

AI responses may gradually shape the author’s writing. Through repeated interaction, such AI-influenced writing may come to feel like the author’s original writing. Once internalized, recognizing the extent of AI contribution may become difficult. Thus, the “pseudo-memory” phenomenon is not entirely new, but AI may amplify and render it more visible. I do not suggest that such AI–human interaction is wrong. I simply note that it may unintentionally prevent authors from making honest disclosure of AI use.

## AI Use Disclosure: For What Purpose?

Let us assume the following case. An author wishes to write an opinion piece in English based on his or her previous Japanese papers. This time, the author has five bullet points to be described. How would editors evaluate the following disclosure? “I drafted five bullet points in Japanese and asked ChatGPT to (1) review my previous publications related to the present theme, (2) incorporate them into this English opinion article, and (3) generate a summary based on them.”

Even if an author had proceeded in this manner, it is unlikely that such a detailed disclosure would ever be made. In practice, statements such as “I used AI for linguistic editing” encompass a wide range of involvement. Some authors may adopt only a single word suggested by AI, whereas others may substantially revise the majority of the manuscript. The phrase “linguistic editing” does not convey the degree of reliance.

AI tools are now ubiquitous. Nonpermissible uses are unlikely to be declared voluntarily. Ultimately, the determination of whether a specific use is permissible rests largely with individual authors. Likewise, the extent of disclosure depends on personal judgment. The pseudo-memory phenomenon described in the previous section may unintentionally hinder accurate self-reporting of AI involvement. Under these circumstances, editorial evaluation will likely focus on the submitted work itself, rather than on the declared presence or absence of AI use.

Taken together, the value of disclosure may therefore lie elsewhere. Rather than directly determining acceptance or rejection, AI disclosure may serve as a record for future examination. If reliable tools for identifying AI influence emerge, the relationship between AI assistance and manuscript quality—or its broader impact on the scientific enterprise—could be systematically studied. The principal value of detailed disclosure may reside in this long-term perspective.

## A Small Wish for Journals

This seasoned author hopes that journals might explicitly state two points. The first is that the use or nonuse of AI tools does not affect acceptance or rejection, provided that such use adheres to journal guidelines and is accurately disclosed. This would parallel the practice of many hybrid journals—offering both open access with article processing charges (APCs) and subscription-based publication without APCs—which explicitly state that the payment or nonpayment of APCs does not influence editorial decisions. The second is that disclosure information is accumulated as a resource for future analysis, contributing to a more transparent publication environment. Such clarification could reduce hesitation surrounding disclosure. Transparency, from both authors and journals, would then become an asset to the medical community rather than a source of uncertainty.

## Article Information

### Author Contributions

Shigeki Matsubara: Conceptualization, investigation, and manuscript writing.

### Conflicts of Interest

None

### Declaration of Artificial Intelligence Use

During the preparation of this work, the author asked ChatGPT-5 to linguistically check the manuscript, with a prompt of “edit only grave errors, while preserving my writing style.” No conceptual arguments, interpretations, or structural components of the main manuscript were generated by artificial intelligence (AI). The author takes full responsibility for all content.

This declaration is intentionally described in some detail. When needed, I use AI only for final manuscript checking with a fixed prompt, resulting in minor word-level changes (e.g., singular to plural or “a” to “the”). I do not suggest that this prompt is adequate for all; however, it may, at least to some extent, reduce the ambiguity of AI use. I include this description in the hope that it may contribute to ongoing discussions on AI use in manuscript writing.

### Approval of Institutional Review Board

Not applicable.

### Patient Anonymity

Not applicable.

### Informed Consent

Not applicable.

### Data Availability

Data sharing is not applicable to this article as no datasets were generated or analyzed during this study.
